# Lipid- and sugar-modified endomorphins: novel targets for the treatment of neuropathic pain

**DOI:** 10.3389/fphar.2013.00155

**Published:** 2013-12-13

**Authors:** Pegah Varamini, Istvan Toth

**Affiliations:** ^1^Medicinal Chemistry Department, School of Chemistry and Molecular Biosciences, The University of QueenslandBrisbane, QLD, Australia; ^2^Medicinal Chemistry Department, School of Pharmacy, The University of QueenslandBrisbane, QLD, Australia

**Keywords:** endomorphin, peptide delivery, neuropathic pain, lipoamino acid, glycosylation, lipopeptide, glycopeptide, blood brain barrier

## Abstract

Endomorphins are endogenous opioid peptides that cause potent antinociception in rodent models of acute and neuropathic pain with less undesirable side effects than opioid alkaloids. However, endomorphins are poorly suited to clinical applications because of low membrane permeability and a susceptibility to enzymatic degradation. Glycosylation and lipidation have proven to be two of the most robust approaches for the generation of new therapeutic endomorphin derivatives. Conjugation with lipoamino acids (LAA) confers an amphipathic character to the peptide, which improved interaction between the peptide and the lipid bilayer of the cell membranes, increasing permeability. Glycosylation can also improve peptide stability and blood brain barrier (BBB) transport. It is believed that an endocytotic mechanism (transcytosis) is responsible for the systemic delivery of water-soluble glycopeptides. This review discusses the application of glycosylation and lipidation strategies to improve the drug-like properties of endomorphins. Pharmacologically active endomorphin analogs with less adverse effects are also discussed.

## Introduction

Opioid analgesics such as morphine are among the most commonly used for the treatment of severe pain. Although opioid analgesics are useful for the relief of nociceptive pain, their efficacy against neuropathic pain is limited. Furthermore, they are associated with a range of undesirable side effects such as constipation, respiratory depression, tolerance, and physical dependence, particularly with long-term use. Investigations into new effective treatments for neuropathic pain that could replace opioid alkaloids have predominantly focused on the development of peptide analogs with selectivity for μ-opioid (MOP) receptors (Vaccarino and Kastin, 2000). In order to be effective as an analgesic for clinical application, the peptide analog must confer high bioavailability, which is achieved through good blood brain barrier (BBB) permeability and resistance to enzymatic degradation. Delivery of pharmaceutical agents to the brain is highly challenging (Banks et al., 1992). Various properties combine to make the BBB a formidable barrier, including tight junctions, minimized surface area, electrostatic interactions and increased metabolism, as well as an active efflux system. Different approaches have been used to improve the brain penetration of pharmaceutical agents (Banks and Kastin, 1985; Begley, 1996). Endomorphin-1 and -2 are naturally occurring peptides with excellent therapeutic potential as replacements for morphine-like opioids. They are potent, highly selective MOP receptor agonists with remarkable anti-neuropathic properties in different rodent models of neuropathic pain (Przewlocki and Przewlocka, 2001) and cause less adverse effects than opioid alkaloids (Vaccarino et al., 1999; Czapla et al., 2000). Nevertheless, like most peptide neurotransmitters and neuromodulators in the CNS, modifications are required to transport endomorphins to the brain.

## Historical perspective

**Figure 1:**
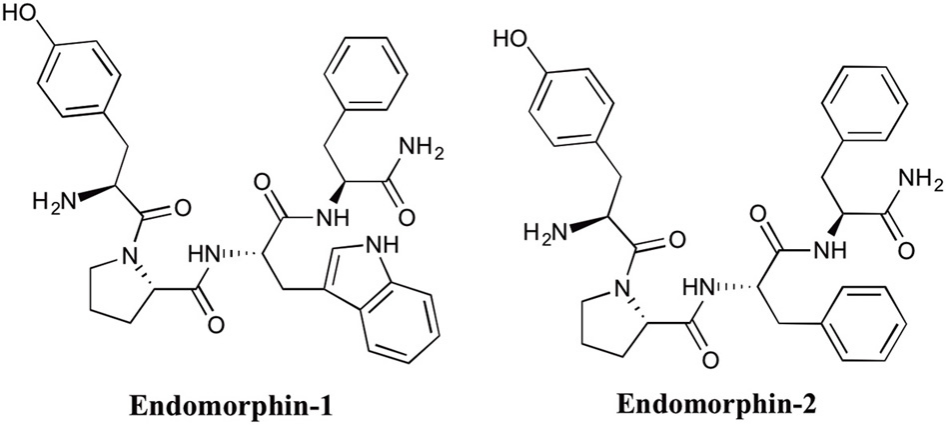
**The structure of endomorphin-1 and -2**.

All endogenous opioid peptides that contain Tyr-Gly-Gly-Phe (including endorphins, enkephakins, and dynorphins), possess affinity for the three opioid receptors: μ (MOP), δ (DOP), and κ (KOP), with low to moderate specificity. β-endorphins have comparable affinity for both MOP and DOP receptors. Met- and leu-enkephalins are DOP receptor endogenous ligands and dynorphins are ligands of KOP receptors. Mammalian peptides with high selectivity and affinity for MOP receptors were not known until the discovery of endomorphins. In 1997, Zadina et al.. replaced the Gly in the endogenous peptide sequence (Tyr-Pro-Trp-Gly-NH_2_) which had high MOP receptor selectivity, but low affinity (Hughes et al., 1975),with all possible natural amino acids (Zadina et al., 1997). Among all derivatives, the Phe-substituted sequence showed the highest affinity and selectivity for MOP receptors and was called endomorphin-1 (Tyr-Pro-Trp-Phe-NH2). Endomorphin-1 and -2, (Tyr-Pro-Phe-Phe-NH2) (Figure 1) were first isolated from bovine brain (Zadina et al., 1997) and then from human cortex (Hackler et al., 1997). Although the precursor(s) of endomorphins remain unidentified, their extraordinarily high affinity and selectivity for MOP receptors in the brain supports the proposal that they are endogenous MOP receptor ligands.

## Strategies to improve bioavailability

Different strategies have been investigated for the effective delivery of endomorphin.

Use of peptidase inhibitors such as tripeptides diprotin A and B, Tyr-Pro-Ala-NH2 (EMDB-2), and Tyr-Pro-Ala-OH (EMDB-3), enhanced and prolonged the antinociceptive effects of endomorphins (Sakurada et al., 2003; Cravezic et al., 2011). However, due to the need for central administration of both peptides and peptidase inhibitors (Fichna et al., 2010; Cravezic et al., 2011), this approach achieved a low degree of success.

Several strategies have been developed to manipulate the structure of endomorphins. The current data indicate that is conformational adaptation of the neuropeptides to the different MOP, DOP, and KOP receptor topographies. Thus, the design of conformationally restricted analogs is of great importance for the selective targeting of a single distinct receptor type (Schiller and DiMaio, 1982). The greater rigidity of the bioactive peptide epitope affects the receptor binding affinity in a receptor-specific way (Bock et al., 2013). Therefore, selectivity of the peptide can be tuned based on the level of conformational restriction imposed by the constraints (Clark et al., 2010).

Glycosylation and lipidation are two successful strategies where peptide conformation is locally restricted. The incorporation of lipids and sugars as conformational constraints (Hruby et al., 1990; Kawai et al., 1990; Hruby and Balse, 2000) significantly improved the pharmacological properties of various peptides (Polt et al., 1994; Egleton et al., 2000; Falconer and Toth, 2007; Cros et al., 2011).

### Glycosylation

**Figure 2:**
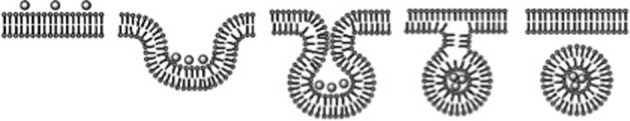
**Endocytosis of glucopeptides (Polt, 2008)**.

Conjugation of carbohydrates to peptides increased the biodistribution of opioid peptides by enhancing permeation across intestinal epithelium and BBB via natural transporters (Poduslo and Curran, 1994; Egleton and Davis, 2005). Membrane proteins such as glucose transporters, GLUT and SGLUT, allowed for the uptake of specific carbohydrates through active, or facilitated transport pathways to deliver peptides across biological barriers (Wood and Trayhurn, 2003). In spite of difficulties associated with their synthesis (Buskas et al., 2006), carbohydrates improved the water solubility, stability and bioavailability of the peptide analogs (Albert et al., 1993; Polt et al., 1994; Negri et al., 1999; Bilsky et al., 2000; Egleton et al., 2000). Although glycosylation reduces passive diffusion of the peptides by reducing their lipophilicity, it makes the peptide a favorable substrate for glucose transporters. Thus, this modification often results in increased penetration through the BBB and GI membranes (Horvat, 2001; Gentilucci, 2004). However, the exact mechanism through which glycosylation improves BBB or GI transport is yet to be elucidated. Octanol/saline distribution studies indicated that transport does not occur via passive diffusion, due to significantly lower lipophilicity (Egleton et al., 2000). Alternatively, the amphipathic nature of glycopeptides was suggested to be responsible for their enhanced permeability through barriers particularly BBB (Palian et al., 2003). For the glycopeptides to be effectively delivered to the brain, it is necessary to produce “biousian” activity (Polt et al., 2005a). *Ousia* means “essence” in Greek. It is important for the glycopeptides to have two essences, an amphipathic state that promotes adsorption to biological membranes and a random coil state that is water-soluble. Biousian effect enabled the compound to undergo endocytosis or permits “membrane hopping” (Egleton et al., 2005). Through extensive studies on a library of glycopeptides, negative membrane curvature on the surface of endothelial cells was shown to be promoted by permeable glycopeptides (Dhanasekaran et al., 2005). This in turn led to an increase in BBB transport (Figure 2) (Broadwell et al., 1988; Egleton et al., 2001; Polt et al., 2005b).

Distribution and pharmacodynamic of the peptides are immensely affected by glycosylation. This allows glycosidic moieties to be used as vectors for targeting specific carbohydrate-recognition receptors (Eduardo, 1994).

### Lipidation

**Figure 3:**
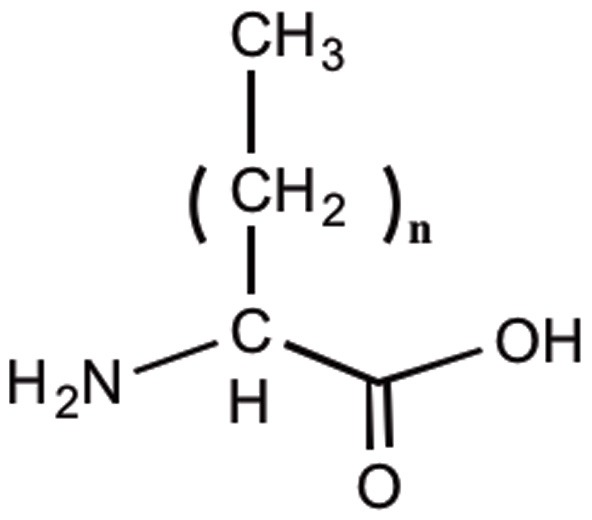
**Structure of lipoamino acids**.

Lipidation is a post-translational peptide modification that significantly influences the properties of peptides and is used in the design of peptide drugs. The presence of polar groups reduced the peptides' partition coefficients and subsequently decreased their membrane permeability (Chikhale et al., 1994). Lipidation provided a simple way to modulate peptide lipophilicity, and facilitates their interaction with cell membranes and penetration across biological barriers by passive diffusion (Balaz, 2000; Griffin and O'Driscoll, 2011). Through increasing the membrane-like properties of the peptides, lipidation improved their interaction with the lipid bilayer within the cell membrane (Pignatello et al., 2005). Both lipoamino acids (LAA) and fatty acid chains have been attached to the peptides to enhance their permeability across biological membranes (Desino et al., 2009). LAAs are α-amino acids with varying length (usually C8–20) alkyl side chains (Figure 3). Having both the hydrophobic properties of lipids and the hydrophilic characteristics of α-amino acids, LAAs are appropriate conjugates to incorporate into the structure of peptides (Toth, 1994; Kokotos et al., 1996). Although the conjugation of fatty acids to the peptides will ultimately result in an increase in their lipophilicity, the addition of LAAs is more advantageous due to their amphipathic character (Toth, 1994). In addition it plays an important role in enhancing peptide's stability against enzymatic degradation (Wang et al., 2006). This in turn affects the absorption, distribution, metabolism and excretion (ADME) and bioavailability of drugs and makes it an attractive strategy to convert peptides into drug leads (Silvius, 2002).

## Physiological and pharmacological properties of lipo- and glyco-endomorphins

### Metabolic stability and membrane permeability

#### Lipoamino acid modification

The endogenous opioid peptide leu-enkephalin was chemically modified by a lipophilic dimethylmaleic anhydride analog. This analog showed a 12- and 32-fold increase in mouse small intestinal mucosal homogenate and liver homogenate (Wang et al., 2006).

**Figure 4:**
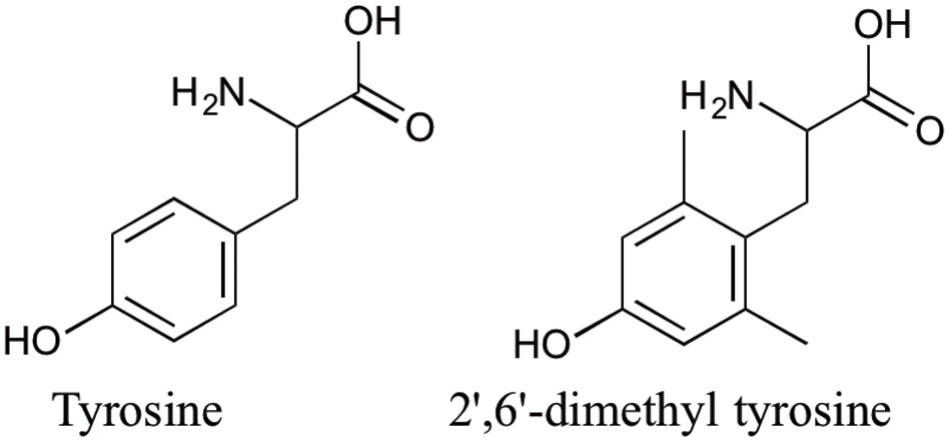
**Structure of Tyrosine and 2′,6′-dimethyltyrosine**.

A series of glycosylated endomorphin-1 peptides were synthesized by modifying either the N- or C-terminus of endomorphin-1 with glucose succinate or glucose, respectively. The half-life of the analog conjugated with glucose at the N-terminus increased from 5 min for endomorphin-1 to 38 min in the Caco-2 cell homogenates. However, the C8LAA-modified glycosylated analog produced even higher stability in the Caco-2 cell homogenate assay with a half-life of 75 min (Koda et al., 2008). Although there was a 3-fold increase in the apparent permeability (*P*_app_) of glucose-C8LAA derivative, this was not as pronounced as the *P*_app_ of the compounds modified only with C8LAA compared to endomorphin-1. Due to a significant reduction in the receptor binding affinity of the C8LAA analog, a further modification was performed on the backbone structure of the parent peptide. The unnatural amino acid 2′,6′-dimethyltyrosine (Dmt) the more hydrophobic and conformationally restricted residue compared to Tyr (Figure 4). Substitution of Tyr with Dmt was shown to enhance the MOP receptor binding affinity and potency of several opioid peptides (Li et al., 2005) including endomorphin-1 (Jinsmaa et al., 2006). Therefore, a Dmt analog, C8LAA[Dmt^1^]endomorphin-1 was synthesized which preserved MOP receptor binding affinity (0.08 nM) relative to the parent peptide. This analog exhibited enhanced stability and permeability (Koda et al., 2008). In another trial, the 10-carbon LAA modified peptides with/without substitution of Tyr^1^ with Dmt^1^ were examined for their biological activity. These endomorphin-1 analogs produced 2–3.5 times higher stability and 2–2.6 times higher permeability for Tyr^1^ and DMT^1^ analogs, respectively, in comparison with their corresponding C8 derivatives (Varamini et al., 2012b).

**Figure 5:**
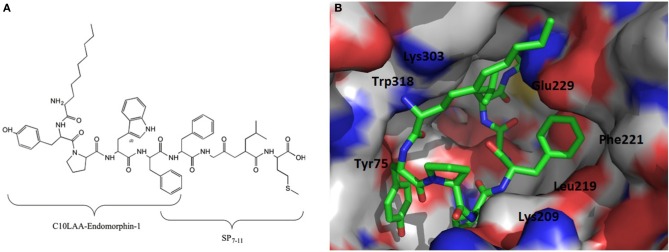
***In vitro* biological analyses revealed that the C10LAA-modified analog conjugated with SP_7−11_ fragment was the most promising derivative. (A)** Structure of the compounds. **(B)** Surface view of the active site of the MOP receptor for the highest docking score conformation of MOP receptor with the compound docked into the active site. For clarity, color of the atoms are as follows: blue—nitrogen, red—oxygen, white—carbon (mu-opioid receptor), and green—carbon (on ligand) (Varamini et al., 2012a).

Substance P is a neuropeptide, which was first reported to elicit analgesic activity in 1976 by Stewart et al. (Stewart et al., 1976). Previously, a hybrid alkaloid/peptide compound, comprised of morphine sulpfate bound to SP_3−11_, produced a strong antinociceptive response with little or no development of opioid tolerance or dependence (Kream, 2003; Kream et al., 2007). C10LAA hybrid endomorphin-1 peptides were designed and synthesized by conjugation of the last 4 or 5 C-terminal amino acids of substance P (SP_8−11_ and SP_7−11_) with all compounds bearing an overlapping Phe in the sequence. It was proposed that the addition of substance P fragments to lipo-endomorphin-1 may decrease the development of tolerance and physical dependence. Two C10-modified endomorphin-1/SP hybrid peptides showed significantly improved half-life and membrane permeability. However, of the two promising LAA-modified derivatives with high stability and permeability, only the one that contained the SP_7−11_ fragment produced potent activity at MOP receptors with significant binding affinity and retained selectivity. The docking scores obtained from conformational studies were also in agreement with the *K*_*i*μ_ values obtained in the receptor binding affinity experiments (Figure 5) (Varamini et al., 2012a).

#### Glycosylation

**Figure 6:**
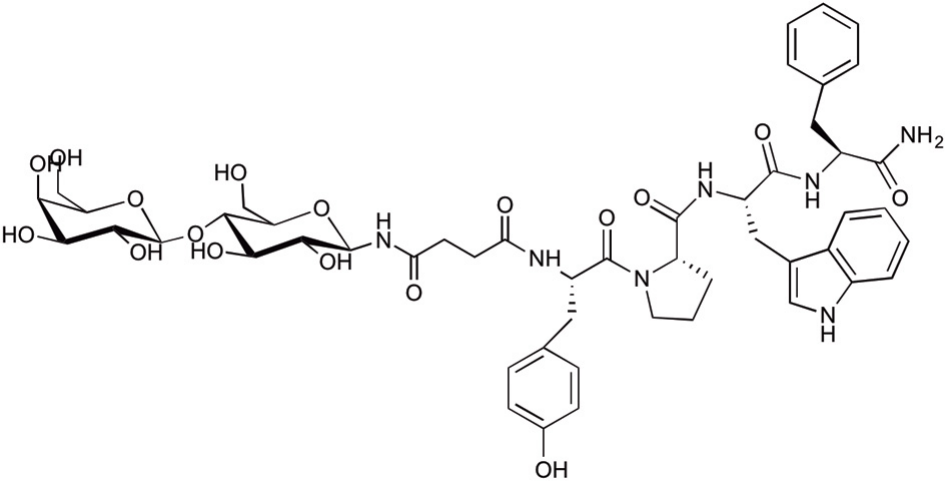
**Structure of lactose succinamic acid-conjugated endomorphin-1**.

A sugar-modified derivative of endomorphin was synthesized by attachment of lactose succinamic acid to the peptide (Figure 6). This glycosylated analog produced an unprecedented 700-fold increase in membrane permeability and 21-fold increase in plasma stability relative to the native peptide (Varamini et al., 2012c). In this study a receptor-mediated or lactose selective transporter-mediated absorption was suggested for as the mechanism of transport across Caco-2 cell monolayer. According to the Biopharmaceutics Classification System, there is a high correlation between Caco-2 cell permeability coefficients (*P*_app_ values) and fractional absorption values (Fa) in humans. This makes the results from Caco-2 cell studies a reliable predictor of the oral absorption of compounds in human (Smetanova et al., 2011). Based on this classification, lactose-endomorphin-1 was considered an ideal candidate for oral delivery.

Opioid peptides are predominantly cleared from the body via fecal-oral routes (Weber et al., 1992; Witt et al., 2001). It has previously been shown that shifting from hepatic to renal clearance can increase plasma stability and improve the antinociceptive effects of opioid peptides (Witt et al., 2001). Since hydrophilicity of the glycopeptides is increased compared with the parent peptides, it is plausible that glycosylation shifts elimination to the renal pathway, thus contributing to the higher stability of these conjugates.

### *In vivo* antinociceptive activity

#### Lipoamino acid modification

Targeting of neuropeptide drugs to the CNS through systemic and oral delivery routes is a formidable obstacle. The delivery of peptide drugs is limited by their poor bioavailability to the brain due to low metabolic stability, high clearance by the liver, and the obstacle posed by the BBB (Egleton et al., 2001).

The 8- and 10-carbon LAA derivatives of endomorphin-1, with either Tyr or Dmt at position 1, have been shown to produce significant dose dependent analgesia in a chronic constriction injury (CCI) model of pain in rats. This analgesia was opioid receptor-mediated and was produced following intravenous (i.v.) administration. The two C10LAA-modified endomorphin-1 peptides produced higher potency than C8LAA analogs and even morphine in this model of rats with ED_50_ values of about 1 μmol/kg. However, no significant difference was observed between ED_50_ values obtained for lipo-endomorphin-1 analogs with Tyr^1^ as in the native form of the peptide, or Dmt^1^ in the modified form. Although C8-Endo-1 and C8-Dmt-Endo-1 (Koda et al., 2008) were reported to have higher MOP receptor binding affinity and agonist activity than their corresponding C10-peptides, their potency in relieving neuropathic pain in CCI rats was less than C10-analogs. The higher antinociceptive potency of C10-modified peptides compared to C8-modified analogs was explained by the increase in their permeability and metabolic stability. Longer alkyl chain length possibly increased analgesic activity by permitting improved transport across lipophilic membranes including the BBB (Varamini et al., 2012b). Furthermore, these lipidated analogs did not cause significant respiratory depression (Varamini et al., 2013), or constipation, and resulted in less tolerance than morphine at analgesic doses (Varamini et al., 2012b). The effect of attaching LAA residues containing different length of alkyl side chain to drug molecules with *in vitro* monoamine oxidase inhibitory activity was also investigated. Consistently, analogs with different LAAs produced significantly different potencies (Pignatello et al., 2005). It is plausible that the divergence in the antinociceptive and side effect profiles of morphine and the lipidic endomorphin-1 derivatives may be due to their different interactions with pharmacologically defined MOP receptor subtypes (Dworkin et al., 2007). Opioid receptor hetero-oligomerization was suggested to be responsible for the diverse pharmacology displayed by these compounds (Jordan and Devi, 1999; Levac et al., 2002).

#### Glycosylation

To date, many glycosylated analogs of various neuropeptides such as deltorphin (Negri et al., 1999), Met-enkephalin (Polt et al., 1994; Egleton et al., 2000), and Leu-enkephalin (Bilsky et al., 2000) have shown improved analgesic activity after peripheral administration. Structure-activity studies of enkephalin-based opioid glycopeptides revealed that disaccharide derivatives were significantly more potent than any of the monosaccharides after peripheral administration (Elmagbari et al., 2004). However, there are only limited reports of the characterization of endomorphin glycopeptides because only few potent derivatives have been developed so far. Biondi *et al.* synthesized glycosylated endomorphin-2 analogs by conjugating glucose or 2,3,4,6-tetra-O-acetyl glucose with the hydroxyproline (Hyp) residue. The MOP receptor binding affinity and agonist activity was abolished in all of these analogs therefore the compounds were not further investigated for their *in vivo* pain relieving activity (Biondi et al., 2006).

N-terminal conjugation of endomorphin-1 with lactose succinamic acid resulted in a significant antinociception following oral administration to CCI rats. This effect was comparable with that of morphine, for which various oral dosage forms are currently in clinical use (Varamini et al., 2012c). In contrast to morphine, the pain-relieving effect of the lactose-endomorphin-1 analog was selective to the injured hindpaw with insignificant effects produced in the contralateral hindpaw. This effect was antagonized by naloxone, which indicated the key role of opioid receptors (Varamini et al., 2012c).

## Conclusion

Endomorphins have been shown to elicit a potent pain relieving effect in different acute and neuropathic pain models in animals after central administration. More importantly this effect is concomitant with little to no undesirable side effects associated with the application of opioids like morphine. These promising effects have been strong motivation for investigators to design and synthesize a substantial number of endomorphin derivatives. Numerous studies report developments in our understanding of the structure activity relationship properties, bioactive conformation, physiological characterizations, *in vivo* and *in vitro* biological activity of endomorphin analogs. Over fifteen years since the discovery of endomorphins, investigations have led to the production of derivatives with acceptable selectivity and MOP receptor-binding affinity. However, only limited progress has been made in the production of compounds with outstanding metabolic stability and membrane permeability, while retaining their pharmacodynamic properties. These are critical criteria in the field of peptide drug delivery to overcome the obstacles and succeed in the development of peripherally active and BBB-permeable analgesics suitable for clinical applications. Thus far, two of the most successful strategies have been shown to be glycosylation and lipid modification. Two examples are the development of an orally active lactose-modified and systemically effective LAA-conjugated derivative of endomorphin-1 with high potential for the treatment of neuropathic pain.

This review highlights the two modifications that have made the most improvements to the therapeutic and side effect profile of endomorphins. Presently potent and promising lead compounds have been developed which are prospective to proceed from research to the pharmaceutical industry. These achievements are the outcome of extensive research having been made to develop opioid peptide-based analgesics like endomorphins for the effective management of neuropathic pain.

### Conflict of interest statement

The authors declare that the research was conducted in the absence of any commercial or financial relationships that could be construed as a potential conflict of interest.
